# Influence of the Environment on Participation in Social Roles for Young Adults with Down Syndrome

**DOI:** 10.1371/journal.pone.0108413

**Published:** 2014-09-26

**Authors:** Kitty-Rose Foley, Sonya Girdler, Jenny Bourke, Peter Jacoby, Gwynnyth Llewellyn, Stewart Einfeld, Bruce Tonge, Trevor R. Parmenter, Helen Leonard

**Affiliations:** 1 Telethon Kids Institute, University of Western Australia, Perth, Australia; 2 School of Occupational Therapy and Social Work, Curtin University, Perth, Australia; 3 Faculty of Health Sciences, University of Sydney, Sydney, Australia; 4 Brain and Mind Research Institute, University of Sydney, Sydney, Australia; 5 Centre for Developmental Psychiatry and Psychology, Monash University, Melbourne, Australia; 6 Sydney Medical School, University of Sydney, Sydney, Australia; 7 Department of Developmental Disability Neuropsychiatry, School of Psychiatry, The University of New South Wales, Sydney, Australia; Vanderbilt University, United States of America

## Abstract

**Background:**

The concept of disability is now understood as a result of the interaction between the individual, features related to impairment, and the physical and social environment. It is important to understand these environmental influences and how they affect social participation. The purpose of this study is to describe the social participation of young adults with Down syndrome and examine its relationship with the physical and social environment.

**Methods:**

Families ascertained from the Down syndrome ‘Needs Opinion Wishes’ database completed questionnaires during 2011. The questionnaires contained two parts, young person characteristics and family characteristics. Young adults’ social participation was measured using the Assessment of Life Habits (LIFE-H) and the influences of environmental factors were measured by the Measure of the Quality of the Environment (MQE). The analysis involved descriptive statistics and linear and logistic regression.

**Results:**

Overall, participation in daily activities was higher (mean 6.45) than in social roles (mean 5.17) (range 0 to 9). When the physical and/or social environment was reported as a facilitator, compared to being no influence or a barrier, participation in social roles was greater (coef 0.89, 95%CI 0.28, 1.52, coef 0.83, 95%CI 0.17, 1.49, respectively). The relationships between participation and both the physical (coef 0.60, 95% CI −0.40, 1.24) and social (coef 0.20, 95%CI −0.47, 0.87) environments were reduced when age, gender, behavior and functioning in ADL were taken into account.

**Conclusion:**

We found that young adults’ participation in social roles was influenced more by the physical environment than by the social environment, providing a potentially modifiable avenue for intervention.

## Introduction

According to the World Health Organisation (WHO), the physical, social and attitudinal factors are important aspects of the environment in which people live and conduct their lives [1]. The experience of disability has been described as an outcome of the interaction between a person’s health or functional impairment and environmental factors. It is now recognised that characteristics of the impairment as well as social and physical factors are important to consider in the understanding of disability [2].

The International Classification of Functioning, Disability and Health (ICF) provides an internationally recognized framework for describing health conditions, health-related states and health outcome measurement [1]. Components of the ICF include body functions and structures, activity, participation and contextual components which include environmental and personal factors. When we recently reviewed factors affecting the transition from school to post-school for young adults with intellectual disabilities we found little information on the impact of environmental factors [3]. Our review employed the ICF as a guiding framework and demonstrated that the ICF is a useful tool for framing transition research.

A large scale longitudinal study investigating influence of environmental factors on participation and quality of life of children and adolescents with cerebral palsy across nine European regions has been undertaken by the SPARCLE group [4], [5]. Levels of participation for children with cerebral palsy were considerably lower than that of the general population and particularly so for those with severe motor impairment and more impairments in general. The attitudinal environment, reflected by environmental law, regulation and physical and social environment, also varied considerably across the European Union countries included in the study [6]. The SPARCLE group employed the Measure of the Quality of the Environment (MQE), which identifies factors that are facilitators or barriers to participation [7]. It is now recognised that measures of the environment should include not only assistive technology and access to and availability of services but also other factors such as access to benefits, friendships and social integration, and attitudes of others and social inclusion [8]. The Measure of the Quality of the Environment (MQE) is an instrument which includes domains addressing these additional factors and can be matched to the ICF categories [9]. Additionally, the MQE domains can be matched to the domains of the Assessment of Life Habits (LIFE-H) a measure of social participation used by the SPARCLE group [5], [10].

The contribution of environmental factors to the disability of individuals with Down syndrome has not been explored previously. Once these contextual factors have been identified, there may be scope to modify them and therefore lessen the experience of disability for young people with Down syndrome and for those with similar intellectual impairments [2]. Therefore, the aims of this research were to use a population-based data source to describe the social participation of young adults with Down syndrome from a parental perspective and to explore the relationship between levels of social participation and the physical and social environment.

## Methods

In 2011 parent report questionnaires were administered to families of young people aged between 16 and 32 years in the Western Australian Down syndrome ‘Needs Opinions Wishes’ (NOW) population-based database [11], [12]. Paper copies of the questionnaires were mailed to families in the Down syndrome NOW database and families were given the option to complete the questionnaire on paper, on the internet or via phone interviews. All families were phoned within a few days of sending out the questionnaires in order to achieve personal contact, provide clear explanation of the study and encourage families to participate. Prior to mailing questionnaires to participants all families were sent a summary booklet of the findings from the previous wave of questionnaires administered in 2009 [11], [13].

The parent report questionnaires contained two parts; part one described young person characteristics including demographic information, presence of medical conditions, health service use and emotional and behavioural problems, as well as information about everyday functioning in activities of daily living, social relationships and day occupations. Part two contained information about family characteristics including family communication, support, informal assistance needs, availability of time and family quality of life. Detailed description of data collection methods has been previously reported [11], [12]. Ethics approval for this study was obtained through the Ethics Committee of the Women’s and Children’s Health Services in Western Australia (Registration number 1715/EP).

### Measures

#### Participation: Assessment of Life Habits (LIFE-H)

The Assessment of Life Habits (LIFE-H) is a measure of social participation and includes twelve life domains (nutrition, fitness, personal care, communication, housing, mobility, responsibilities, employment, education, relationships, community life and recreation). It is also possible to calculate daily activities and social roles accomplishment sub scores [14]. The LIFE-H has been employed in populations of people with spinal cord injury, stroke, traumatic brain injury, children and older adults with cerebral palsy [15]–[17]. The scores can also be presented in accordance with the ICF, by quantifying the scores by percentiles and applying the appropriate qualifying words. For example, minor restrictions (LIFE-H score ≥8), moderate (LIFE-H score 4–7) or severe restrictions (LIFE-H score ≤3). This provides a universally understood and clinically relevant presentation of the data [18].

Two specific elements are involved in this measure, 1) level of accomplishment of the daily activity and 2) type of assistance required (no assistance, adaptation, device or human assistance). An item score between 0 (not accomplished) to 9 (accomplished independently, without difficulty) is calculated for each life domain (scoring key is shown in Table 1). In order to account for the variable number of items within each domain of life habits and the ‘non applicable’ items, a scoring system has been proposed [16], [19]. A weighted score was calculated by the summation of raw scores, divided by the number of applicable items [16], [19]. A score may be obtained for each item, each life domain (mean of items), or for the two subscales (daily activities and social roles). We did not include the education life domain in the social roles sub-score, as many participants had already left school and thus this life domain was only applicable to less than half of the sample (n = 80). Parent reported level of satisfaction was scored within each life domain and was reported on a 5-point likert scale 0 (very dissatisfied) to 4 (very satisfied). The satisfaction score is reported separately and used to evaluate the individual’s quality of social participation [14].

**Table 1:** Life habits accomplishment scale.

Score	Difficulty level	Assistance type
9	No difficulty	No assistance
8	No difficulty	Assistive device (or adaptation)
7	With difficulty	No assistance
6	With difficulty	Assistive device (or adaptation)
5	No difficulty	Human assistance
4	No difficulty	Assistive device (or adaptation) and human assistance
3	With difficulty	Human assistance
2	With difficulty	Assistive device (or adaptation) and human assistance
1	Accomplished by a proxy	
0	Not accomplished	
NA	Not applicable	

#### Environment: Measure of the Quality of the Environment (MQE)

The MQE was designed to identify environmental factors which were facilitators or barriers to participation and has been used to measure their influence on people with stroke, cerebral palsy and spinal cord injuries [7], [20]–[25]. The items correspond to the environmental factors described within the ICF [20] and cover six domains: social support and attitudes (14), income, labour and income security (15), government and public services (27), equal opportunities and political orientations (10), physical environment and accessibility (38) and technology (5). Generally the last two domains refer to the physical environment (40 items) while the remainder refer to the social environment (69 items) [7].

#### Emotional and behavioural problems: Developmental Behaviour Checklist – Adult Version (DBC-A)

The DBC-A is 107-item checklist which measures emotional and behavioural problems and was developed specifically for use with adults with intellectual and/or developmental disability. Each behavioural response is scored as 0 (not true as far as you know), 1 (somewhat or sometimes true) or 2 (very true or often true). The DBC-A has been found to have acceptable test-retest and inter-rater reliability and convergent ability has been demonstrated with two measures of behavioural disturbances of adults with intellectual disability [26].

#### Functioning in activities of daily living: Index of Social Competence (ISC)

The Index of Social Competence (ISC) [27] was used to measure domains of communication, self-care and community skills. This measure discriminates well between different levels of ability [28].

### Data Analysis

Descriptive statistics including means, standard deviations and ranges, were used to describe the participation (LIFE-H) and environment (MQE) data. Univariate relationships between independent variables and the outcome, subscores of the LIFE-H, were examined using analysis of variance and chi-squared tests. Logistic regressions with binary outcomes were used in the final models allowing for adjustment for confounding variables. The outcome was binary as we combined those who reported the environment as having ‘no influence’ or being a ‘barrier’ together and compared them to those who reported the environment as a facilitator. It is useful to identify those environmental factors which are facilitators to then provide targets for intervention to make a positive impact on participation for young people with Down syndrome. Confounding variables were identified through the use of the ICF. Examining the relationship between participation and environment required accounting for confounding variables which represented the other domains of the ICF. Therefore the confounding variables which were adjusted for in the final model were age and gender (personal factors), emotional and behavioural problems (impairment of body functions and structures) and functioning in activities of daily living (activity) [1]. Unadjusted and adjusted models were reported separately. STATA 11 was used for all analyses [29].

## Results

Families of 197/223 (88.3%) young people returned the 2011 Down syndrome ‘NOW’ questionnaire. This study will focus on the 166/197 (84.3%) families who returned the parent report questionnaires with sufficient data on the participation and environment measures. The majority (136/166, 81.9%) of the young adults lived with their parents in their family home, others lived with other family or friends (11/166, 6.6%), five lived in a group home (3.0%) and four young adults lived alone (2.4%).

### Participation

Eight (4.9%) young adults were reported by their parents as experiencing severe restrictions in participation in daily activities, 126 (75.9%) moderate and 27 (16.3%) minor restrictions. Participation in social roles was reported as severely restricted in 18 young adults (10.8%), moderate for 117 (70.5%) and a minor restriction for six (3.6%)(Table 2). The domain reported with the lowest participation score was the responsibilities domain (mean 3.75 SD 2.27), which relates to recognizing the value of money, making purchases and planning budgets. Participation in education (mean 4.52, SD 2.67), community life (mean 4.72 SD 2.54) and recreation (mean 4.81, SD 2.38) also scored low participation scores (Table 2). These are all domains normally included in the social roles subscore but for this study we did not include the education domain. Participation in housing and fitness domains scored the highest of the domains within the LIFE-H (mean 7.51 SD 1.69, mean 7.41 SD 1.66, respectively). The housing domain involved taking part in housekeeping tasks, entering and exiting the home and using household equipment (furniture, lighting and outdoor equipment). The fitness domain described participating in physical activities and relaxation activities as well as sleep and getting in and out of bed.

**Table 2:** Mean LIFE-H scores by life domain categories and sub-scores (Daily activity and Social roles) and number of participants reported as experiencing severe, moderate or minor restrictions in participation.

				Participation Restrictions			
Continuous variables	Frequency	Mean LIFE-H score (SD)	Range LIFE-H score	Severe n (%) (score ≤3)	Moderate n (%) (score 4–7)	Minor n (%) (Score ≥8)	Missing/NA n (%)
Social Participation (LIFE-H)							
*Daily activities categories*							
Nutrition	166	5.91 (2.14)	0.25, 9	18 (10.8)	112 (67.5)	36 (21.7)	0
Fitness	164	7.41 (1.66)	1.25, 9	2 (1.2)	81 (48.8)	81 (48.8)	2 (1.2)
Personal care	165	6.33 (2.29)	0, 9	20(12.0)	99 (59.6)	46 (27.7)	1 (0.6)
Communication	164	5.60 (2.48)	0, 9	29 (17.5)	99 (59.6)	36 (21.7)	2 (1.2)
Housing	162	7.51 (1.69)	0.5, 9	4 (2.4)	80 (48.2)	78 (47.0)	4 (2.4)
Mobility	163	5.90 (2.11)	0.2, 9	15 (9.0)	113 (68.1)	35 (21.1)	3 (1.8)
Daily activities subscore	161	6.45 (1.65)	0.81, 8.86	8 (4.9)	126 (75.9)	27 (16.3)	5 (3.0)
*Social roles categories*							
Responsibility	161	3.75 (2.27)	0, 9	68 (41.0)	87 (52.4)	6 (3.6)	5 (3.0)
Employment	146	5.47 (2.83)	0, 9	34 (20.5)	76 (45.8)	36 (21.7)	20 (12.0)
Education a	80	4.52 (2.67)	0, 9	37 (22.2)	27 (16.3)	16 (9.6)	86 (51.8)
Relationships	159	6.66 (2.34)	0, 9	17 (10.2)	75 (45.2)	67 (40.4)	7 (4.2)
Community life	163	4.72 (2.54)	0, 9	52 (31.3)	87 (52.4)	24 (14.5)	3 (1.8)
Recreation	159	4.81 (2.38)	0, 9	44 (26.5)	93 (56.0)	22 (13.3)	7 (4.2)
Social roles subscore	141	5.17 (1.84)	0.22, 8.93	18 (10.8)	117 (70.5)	6 (3.6)	25 (15.1)

a Education not included in social roles subscore.

### Environment

Parent perception of whether the environmental factor within the MQE was a major obstacle or major facilitator was scored on a 7-point Likert scale. Reponses to each item are presented in Table 3. Two continuous scores were calculated by summing the items and dividing into percentiles, one describing the physical environment and one describing the social environment. Approximately one third of the parents who provided data on the MQE reported the social environment as mainly a facilitator to participation (n = 45, 27.1%) and just over one third reported the physical environment as mainly a facilitator (n = 59, 35.5%). Just over one third reported the social and physical environment as having no influence (n = 63, 38.0%, n = 64, 38.6%, respectively) and a very small proportion reported the overall social and physical environment as a barrier to participation (n = 2, 1.2%, n = 7, 4.2%). Data for the remaining families concerning the influence of the overall social and physical environment were missing, reported as ‘I don’t know’ or ‘Does not apply’ (Table 3).

**Table 3:** Parent report influences of environmental factors on the accomplishment of daily activities (n = 166).

Environmental factors	Barriern (%)	No influencen (%)	Facilitatorn (%)	Does not applyn (%)	Missing/I don’t known (%)
*Social environment Subscale*	2 (1.2)	63 (38.0)	45 (27.1)	56 (33.7)	^a^
Social networks					
Family situation	13 (7.8)	6 (3.6)	125 (75.3)	10 (6.0)	12 (7.2)
Support from family	16 (9.6)	12 (7.2)	115 (69.3)	13 (7.8)	10 (6.0)
Support from friends	44 (26.5)	21 (12.7)	70 (42.2)	19 (11.4)	12 (7.2)
Support from neighbours	34 (20.5)	1 (0.6)	45 (27.1)	26 (15.7)	60 (36.1)
Support from colleagues	13 (7.8)	15 (9.0)	107 (64.5)	18 (10.8)	13 (7.8)
Attitudes of people around					
Families and close friends	9 (5.4)	9 (5.4)	120 (72.3)	16 (9.6)	12 (7.2)
Attitudes of friends	15 (9.0)	26 (15.7)	95 (57.2)	13 (7.8)	17 (10.2)
Attitudes of colleagues	7 (4.2)	13 (7.8)	118 (71.2)	11 (6.6)	17 (10.2)
Attitudes of superiors	8 (4.8)	12 (7.2)	121 (72.3)	8 (4.8)	17 (10.2)
Attitudes of neighbours	12 (6.1)	52 (31.3)	65 (39.2)	19 (11.4)	21 (12.7)
Attitudes of service providers	15 (7.2)	20 (10.1)	107 (64.5)	5 (3.0)	22 (13.3)
Attitudes of strangers	26 (15.7)	43 (12.0)	69 (41.6)	8 (4.8)	23 (13.9)
Attitudes of people when there in a group(class, crowd)	22 (13.3)	25 (12.6)	93 (56.0)	5 (3.0)	24 (14.5)
Religious beliefs of people in your community	7 (4.2)	60 (15.1)	53 (31.9)	22 (13.3)	24 (14.5)
Employment services					
Counseling and employment seeking services	13 (7.8)	33 (19.8)	43 (25.9)	55 (33.1)	22 (13,3)
Current availability of jobs in your community	40 (24.1)	26 (15.7)	15 (9.0)	55 (33.1)	30 (18.1)
Job criteria/tests	38 (22.9)	23 (13.9)	13 (7.8)	57 (34.3)	35 (21.1)
Currently employed only					
Their workplace	6 (3.6)	7 (4.2)	77 (46.4)	76 (45.8)	^a^
Requirements of work tasks	7 (4.2)	4 (2.4)	81 (48.8)	74 (44.6)	^a^
Their work hours	6 (3.6)	6 (3.6)	76 (45.8)	77 (46.4)	^a^
Union structures	3 (1.8)	28 (16.9)	15 (9.0)	120 (72.3)	^a^
Employee services	2 (1.2)	17 (10.2)	40 (24.1)	107 (64.5)	^a^
Financial Resources					
Personal income	23 (13.8)	24 (14.5)	93 (56.0)	7 (4.2)	22 (13.3)
Public disability programs (e.g. Disability pensions)	16 (9.6)	16 (9.6)	108 (65.1)	2 (1.2)	22 (13.3)
Private health insurance programs	16 (9.6)	35 (21.1)	68 (41.0)	28 (16.9)	22 (13.3)
Commercial services					
Availability of business (e.g. shopping centres)	12 (7.2)	29 (17.5)	88 (53.0)	18 (10.8)	22 (13.3)
Services offered by business	11 (6.6)	43 (25.9)	66 (39.8)	22 (13.3)	24 (14.5)
Other support services					
Support workers other than family	7 (4.2)	15 (9.0)	105 (63.3)	23 (13.9)	16 (9.6)
Home care services	7 (4.2)	34 (20.5)	40 (24.1)	70 (42.2)	15 (9.0)
Health services (e.g. hospital, medical clinic)	9 (5.4)	25 (15.1)	100 (60.2)	15 (9.0)	17 (10.2)
Physical and social rehabilitation services in community	7 (4.2)	39 (23.5)	38 (22.9)	62 (37.3)	20 (12.0)
Vocational services in community	11 (6.6)	40 (24.1)	30 (18.1)	60 (36.1)	25 (15.1)
Social integration support services(eg social work, residential resources)	14 (8.4)	34 (20.5)	42 (25.3)	51 (30.7)	25 (15.1)
Educational services					
Educational service in community (e.g. TAFE)	2 (1.2)	5 (3.0)	31 (18.7)	7 (4.2)	121 (72.9)
Access to student loans	1 (0.6)	36 (21.7)	5 (3.0)	1 (0.6)	123 (74.1)
Other educational services	1 (0.6)	15 (9.0)	7 (4.2)	21 (12.7)	122 (73.5)
*Physical environment subscale*	7 (4.2)	64 (38.6)	59 (35.5)	36 (21.7)	^a^
Public infrastructure					
Public transport	36 (21.7)	19 (11.4)	57 (34.3)	41 (24.7)	13 (7.8)
Specially routed buses/trains for people with disabilities	29 (17.5)	28 (16.9)	29 (17.5)	67 (40.4)	13 (7.8)
Long distance transport (e.g. bus, plane)	19 (11.4)	45 (27.1)	33 (19.9)	51 (30.7)	21 (12.7)
Communication services (e.g. telephone, internet)	13 (7.8)	33 (19.9)	67 (40.4)	37 (22.2)	16 (9.6)
Radio media services	8 (4.8)	57 (34.3)	37 (22.2)	47 (28.3)	17 (10.2)
Television media services	8 (4.8)	0	98 (59.0)	37 (22.2)	23 (13.9)
Community organization services					
Cultural services	6 (3.6)	18 (10.8)	104 (62.7)	22 (13.3)	16 (9.6)
Religious organizations	6 (3.6)	45 (27.1)	54 (32.5)	45 (27.1)	16 (9.6)
Athletic and recreational organization services	11 (6.6)	19 (11.4)	105 (63.3)	17 (10.2)	14 (8.4)
Community organizations (e.g. craft/social groups)	11 (6.6)	33 (19.9)	68 (41.0)	37 (22.2)	17 (10.2)

a‘ Missing/I don’t know’ data presented with ‘Does not apply’ data column due being unable to distinguish between the two categories for these specific questions.

Within the social environment sub-scale the most commonly reported facilitators to participation were the family situation (n = 125, 75.3%), attitudes of families and close friends (n = 120, 72.3%), colleagues (n = 118, 71.2%) and superiors (n = 121, 72.3%). The most commonly reported barriers to participation in the social environment were related to support from friends (n = 44, 26.5%) and neighbours (n = 34, 20.5%), current availability of jobs (n = 40, 24.1%) and job criteria (n = 38, 22.9%) and attitudes of strangers (n = 26, 15.7%).

### Relationship between participation and physical and social environment

The two subscores of the participation measure, daily activities and social roles, and their relationship with independent variables including demographics, behavior, and environmental factors are presented in Table 4. There was no difference in participation in daily activities or social roles by gender, family income or place of residence. Attitudes of others were associated with participation in daily activities, with those parents who considered attitudes of others to be a facilitator or have no influence reporting a higher participation score (mean 6.33 SD 1.44, mean 6.71 SD 1.50, respectively) than those who considered attitudes of others to be a barrier (mean 4.16 SD 2.08). Similarly, those who considered social networks to be a barrier were more likely to report lower participation in daily activities (mean 5.46 SD 2.01) than those who considered social networks to be a facilitator or have no influence (mean 6.13 SD 1.84, mean 6.69 SD 1.35, respectively). Those who considered the influence of commercial services such as grocery stores, restaurants and shopping centres as a facilitator to participation reported higher participation in social roles (mean 5.53 SD 1.59) than those who considered them as barriers (mean 4.73 SD 1.84). This relationship was weaker for participation in daily activities (Table 4).

**Table 4:** Univariate relationship between social participation and independent variables (n = 166).

	Social Participation (LIFE-H) (0–9)					
	Daily activities sub-score			Social roles sub-score		
Independent variables	Frequency	Mean (SD)	P-value	Frequency	Mean (SD)	P-value
Personal factors						
Gender						
Male	88 (53.0)	6.37 (1.78)	0.50	77 (46.4)	5.02 (1.8)	0.28
Female	73 (44.0)	6.54 (1.49)		64 (38.6)	5.36 (1.88)	
Missing	5 (3.0)	–		25 (15.1)	–	
Age group						
16≤20 year olds	36 (21.7)	6.01 (1.79)	0.11	29 (17.5)	4.79 (1.61)	0.44
21≤25 year olds	52 (31.3)	6.38 (1.72)		46 (27.7)	5.32 (2.01)	
26≤32 year olds	73 (44.0)	6.71 (1.49)		66 (39.8)	5.24 (1.80)	
Missing	5 (3.0)	–		25 (15.1)	–	
Environmental factors						
Annual family income						
$78000 and above	72 (43.4)	6.58 (1.55)	0.47	36 (21.7)	5.22 (1.91)	0.88
Between $41600 and $77999	33 (19.9)	6.18 (1.61)		27 (16.3)	5.04 (1.41)	
Less than $41599	42(25.3)	6.33 (1.81)		65 (39.2)	5.07 (2.03)	
Missing	19 (11.4)	–		38 (22.9)		
Place of residence						
Family home	136 (81.9)	6.32 (1.67)	0.14	120 (72.3)	5.04 (1.81)	0.34
Group home/hostel	5 (3.0)	6.40 (1.46)		4 (2.4)	5.35 (2.16)	
Living alone	4 (2.4)	7.02 (0.80)		3 (1.8)	5.74 (1.71)	
Living with family/friends	11 (6.6)	7.48 (1.53)		9 (5.4)	6.14 (1.99)	
Missing	10 (6.0)	–		30 (18.1)	–	
Living Region						
Major city (Perth)	117 (70.5)	6.42 (1.65)	0.70	102 (61.4)	5.09 (1.79)	0.40
Regional/remote	44 (26.5)	6.53 (1.66)		39 (23.5)	5.38 (1.95)	
Missing	5 (3.0)	–		25 (15.1)	–	
MQE Subscales						
Social networks						
Barrier	12 (7.2)	5.46 (2.01)	0.01	10 (6.0)	4.36 (1.49)	0.14
No influence	42 (25.3)	6.13 (1.84)		34 (20.5)	4.90 (1.79)	
Facilitator	94 (56.6)	6.69 (1.35)		87 (52.4)	5.37 (1.76)	
Missing	18 (10.8)	–		35 (21.1)	–	
Attitudes of others						
Barrier	5 (3.0)	4.16 (2.08)	<0.01	5 (3.0)	4.01 (1.42)	0.24
No influence	52 (31.3)	6.33 (1.44)		43 (25.9)	5.22 (1.59)	
Facilitator	89 (53.6)	6.71 (1.50)		81 (48.8)	5.34 (1.79)	
Missing	20 (12.0)			37 (22.3)	–	
Employment services						
Barrier	19 (11.4)	6.12 ((1.93)	0.21	16 (9.6)	4.66 (1.75)	0.088
No influence	42 (25.3)	6.53 (1.51)		38 (22.9)	5.22 (1.82)	
Facilitator	59 (35.5)	6.80 (1.31)		57 (34.3)	5.67 (1.59)	
Missing	46 (27.7)	–		55 (33.1)	–	
Financial resources						
Barrier	13 (7.8)	5.09 (2.33)	<0.01	9 (5.4)	3.99 (1.56)	0.10
No influence	37 (22.3)	6.48 (1.39)		31 (18.7)	5.41 (1.61)	
Facilitator	92 (55.4)	6.55 (1.55)		85 (51.2)	5.25 (1.85)	
Missing	24 (14.5)	–		41 (24.7)	–	
Commercial services						
Barrier	11 (6.6)	5.55 (2.21)	0.057	8 (4.8)	4.73 (1.84)	0.04
No influence	37 (22.3)	6.34 (1.50)		34 (20.5)	4.69 (1.69)	
Facilitator	76 (45.8)	6.68 (1.39)		70 (42.2)	5.53 (1.59)	
Missing	42 (25.3)	–		54 (32.5)	–	
Other support services						
Barrier	5 (3.0)	4.70 (2.79)	0.04	5 (3.0)	4.08 (1.68)	0.29
No influence	55 (33.1)	6.19 (1.69)		48 (28.9)	5.05 (1.87)	
Facilitator	78 (47.0)	6.58 (1.59)		69 (41.6)	5.31 (1.77)	
Missing	28 (16.9)	–		44 (26.5)	–	
Education services						
Barrier	1 (0.6)	7.78 (0)	0.72	1 (0.6)	6.65 (0.0)	0.62
No influence	18 (10.8)	6.34 (1.47)		15 (9.0)	5.26 (1.87)	
Facilitator	25 (15.1)	6.44 (1.91)		21 (12.7)	4.94 (1.82)	
Missing	122 (73.5)	–		129 (77.7)	–	
Public infrastructure						
Barrier	17 (10.2)	5.67 (2.00)	0.05	14 (8.4)	4.85 (1.70)	0.22
No influence	49 (29.5)	6.55 (1.37)		45 (27.1)	5.12 (1.83)	
Facilitator	61 (36.7)	6.75 (1.69)		55 (33.1)	5.60 (1.66)	
Missing	39 (23.5)	–		52 (31.3)	–	
Community organization services						
Barrier	4 (2.4)	4.34 (1.97)	0.01	3 (1.8)	3.55 (1.72)	0.10
No influence	38 (22.9)	6.34 (1.44)		35 (21.1)	5.01 (1.87)	
Facilitator	98 (59.0)	6.62 (1.56)		85 (51.2)	5.42 (1.61)	
Missing	26 (15.7)	–		43 (25.9)	–	
Physical environment subscore						
Barrier	6 (3.6)	4.33 (1.54)	<0.01	5 (3.0)	3.34 (1.26)	<0.01
No influence	57 (34.3)	6.42 (1.45)		51 (30.7)	5.06 (1.77)	
Facilitator	62 (37.3)	6.90 (1.52)		56 (33.7)	5.80 (1.49)	
Missing	41 (24.7)	–		54 (32.5)	–	
Social environment subscore						
Barrier	2 (1.2)	2.76 (1.13)	<0.01	2 (1.2)	2.77 (1.36)	0.01
No influence	44 (26.5)	6.33 (1.67)		37 (22.3)	4.88 (1.68)	
Facilitator	61 (36.7)	6.76 (1.21)		59 (35.5)	5.60 (1.54)	
Missing	59 (35.5)	–		68 (41.0)	–	
Day Occupation						
Still at school	10 (6.0)	5.68 (2.14)	<0.001	7 (4.2)	4.37 (2.00)	<0.001
Open employment	35 (21.1)	7.15 (1.13)		33 (19.9)	5.99 (1.73)	
Training	20 (12.0)	6.90 (1.25)		18 (10.8)	5.56 (1.28)	
Sheltered employment	61 (36.7)	6.60 (1.47)		59 (35.5)	5.32 (1.57)	
Day recreation program	33 (19.9)	5.30 (1.90)		24 (14.5)	3.65 (2.05)	
Not working	2 (1.2)	7.39 (0.8)		0	–.	
Missing	5 (3.0)	–		25 (15.1)	–	

**Figure 1:**
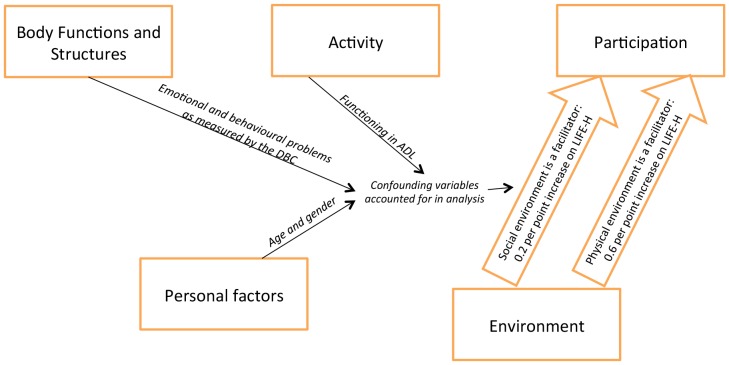
Relationship between social participation and the physical and social environment.

The unadjusted logistic regression model showed that when the physical and/or social environment was considered as a facilitator, compared to being no influence or a barrier, then participation in social roles increased (coef 0.89, 95%CI 0.28, 1.52, coef 0.83, 95%CI 0.17, 1.49, respectively)(Table 5). Confounding variables of age, gender, emotional and behavioural problems as measured by the DBC and functioning in activities of daily living (ADL) as measured by ISC were included in the adjusted regression model (Table 5). The addition of these confounding variables reduced the strength of the relationship between the facilitating effect of the social environment and increased participation in social roles (coef 0.20, 95%CI −0.47, 0.87). This was similar for the physical environment, however the effect persisted more so than for the social environment (coef 0.60, 95% CI −0.40, 1.24). These results are graphically represented in Figure 1. We considered stratifying the regression analysis by level of functioning in order to investigate if there were differences in the participation outcome. Other studies have identified that functioning in activities of daily living can be associated with different domains of participation [11], [30], [31]. However when we explored this interaction in this study within the regression between functioning in ADL and environment no association was found.

**Table 5:** Binary logistic regressions of the relationship between the physical and social environment and participation in social roles.

		Social roles subscale of LIFE-H					
		Unadjusted model*			Adjusted model (n = 93)		
	Freq	Coeff†	95% CI	p-value	Coeff†	95% CI	p-value
Physical environment subscore							
Barrier/No influence		Reference			Reference		
Facilitator	112	0.89	0.28, 1.52	0.005	0.60	−0.40, 1.24	0.06
Social environment subscore							
Barrier/No influence		Reference			Reference		
Facilitator	98	0.83	0.17, 1.49	0.015	0.20	−0.47, 0.87	0.59

Note: Adjusted model includes confounding variables age, gender, emotional and behavioural problems, functioning in ADL.

*The unadjusted models are two separate models, physical environment (n = 112) and social environment (n = 98), whereas the adjusted model is one single model.

† Per point increase on the outcome measure, Social Roles subscale of the LIFE-H.

## Discussion

Young people with Down syndrome were reported to have more difficulty participating in social roles (e.g. relationships, community life, recreation etc.) than they did participating in daily activities (e.g. personal care, communication, housing etc.). The majority of young people with Down syndrome experience moderate participation restrictions in daily activities and social roles. We found that young adults’ participation in social roles was considered from a parental perspective to be influenced by the physical environment (including public infrastructure and community organization services) more than by the social environment, however both were weak associations. Of concern, is the fact that the most commonly reported barriers to participation were negative attitudes of strangers, and lack of support from friends, availability of jobs and public transport. The most commonly cited facilitators to young person participation reported by parents were family and close friends, young person’s current workplace (if they were employed), and attitudes of superiors and colleagues of the young person.

The main strength of this study is that it is framed within the internationally recognized disability framework, the ICF. Examining the complex situation of the environment’s influence on social participation while accounting for personal (age and gender) and impairment factors (emotional and behavioural problems) can be clarified through the use of the ICF. Another strength is the quantitative description through a standardized measure of social participation and the influence of the environment of young people with Down syndrome through the use of cases ascertained from a population-based database [8]. The high response fraction enables further generalization of findings to the wider population of young people with Down syndrome across Australia and internationally and perhaps intellectual disability from other causes. However, there were some missing data in the environment (MQE) and participation (LIFE-H) measures. The cross-sectional design of this study meant we were unable to define the causal direction of the relationship between participation and environment. Another consideration is the fact that the young people may not consider barriers to participation in the same way as their parents. However, a significant strength is that there were barriers to participation identified from the parents’ perspective (for example attitudes of others, availability of jobs and public transport) that have the potential to be modified through policy and intervention strategies.

The finding of a relationship between the physical environment as a facilitator and increased participation in social roles was interesting. We had hypothesized that the social environment would have had a stronger influence on participation in social roles. Elements of the physical environment included public transport, cultural and religious services and recreational and community organization. Previous research into factors that were barriers to social inclusion from the perspectives of young people with an intellectual disability highlighted four main elements, one of which related to the physical environment. This element was the location of their house, and the availability of transport to and from the house [32]. In Ireland, barriers to leisure participation for adolescents with intellectual disability were ‘access to’ and ‘location of’ leisure facilities from both young person and parent perspectives [33]. Inclusion of the variables representing the body functions and structures and activity domains of the ICF reduced the strength of the relationship, further highlighting the complex interaction between the social-psychological and biological factors that contribute to overall functioning. Emerging evidence suggests that the physical environment has the potential to have a large impact on the participation for young adults with intellectual disability and provides a new avenue for intervention.

From the parents’ perspective, our study shows that the attitudes of others act as a barrier to participation for young people with Down syndrome. Previous research involving people with an intellectual disability has explored social distance and described the relative willingness of an individual to take part in relationships of varying degrees of intimacy with a person who has a stigmatized identity [34]. The authors of this research reported that older people and people with lower education levels endorsed a higher level of social distance between themselves and people with an intellectual disability [34]. Research has highlighted that public knowledge of intellectual disability and causal beliefs are particularly under-researched areas and that one of the main reasons for lay people’s reluctance to interact with people with intellectual disability is due to discomfort and anxiety [35]. Clearly, public campaigns which promote education and understanding around people with intellectual disability could play a role in limiting social distance and in turn facilitate participation in social roles for people with Down syndrome.

Workplace characteristics including attitudes of superiors and colleagues and the work environment in general were cited by parents as facilitators to participation for those young people who were employed. A questionnaire study involving 643 Australian employers who had employed a person with a disability found that the person with a disability was reported as better than the ‘average’ employee on reliability variables (attendance and sick leave) and maintenance variables (recruitment, safety, insurance costs) [36]. Also, a Canadian study which surveyed the public on views on employment of people with intellectual disabilities found that the people surveyed believed that employing a person with a disability in a workplace would not have a negative effect on the workplace. However, the respondents did highlight lack of employment training programs for people with intellectual disability as a major obstacle to gaining their employment [37]. People with intellectual disability who participated in focus groups and were asked about their perspective on barriers to social inclusion did not cite being employed as a way to improve their inclusion [38]. However, those who were employed, often mention social inclusion as a valued outcome of participating in employment [39]. While young people with Down syndrome have been reported to find it difficult to find appropriate and suitable jobs [40], [41], it is encouraging that once they were in the workplace, their environment was supportive.

This study has focused on the environmental factors that families reported as facilitators to participation in order to identify avenues for intervention. However, factors reported as barriers to participation are important to consider. Overall, there were very small proportions of families who reported the social or physical environment as a barrier, yet just over one third of families reported that the social and physical environment had ‘no influence’ on their sons/daughters participation. Service providers who are aiming to facilitate increased participation for young people with intellectual disabilities should consider adjusting these existing social and physical environmental factors, which act as no influence, to have a positive influence on participation.

There is a developing body of knowledge which reports the impact which negative community attitudes have on social inclusion for young people with intellectual disability [32]. Reports of increased rates of violence against people with disabilities including intellectual disability is concerning [42]. In the United States it has been reported that those people with an intellectual disability had a higher risk of violent victimization than persons with any other type of disability and those with intellectual disability experience a higher frequency of sexual assault, robbery and aggravated assault than those with a sensory disability [43]. A review involving studies from United States, Australia, England and Spain found higher prevalence of physical and sexual abuse maltreatment towards people with intellectual disability compared to those without intellectual disability [44]. There is an urgent need to address the lack of population-level data which clearly defines this issue, to then effectively guide resource allocation and service delivery [44], [45].

## Conclusion

Through the use of the internationally renowned framework, ICF, this study has highlighted that young people with Down syndrome experience participation restrictions in involvement in social roles. Parents reported that elements of the environment negatively influence participation including negative attitudes of strangers, and lack of support from friends, availability of jobs and public transport. This study has highlighted the important influence of the physical environment on social participation. This is an influence which may have been previously overlooked and has great potential to be modifiable.
